# Hypo-fractionated SBRT for localized prostate cancer: a German bi-center single treatment group feasibility trial

**DOI:** 10.1186/s13014-017-0872-2

**Published:** 2017-08-18

**Authors:** Ping Jiang, Katja Krockenberger, Reinhard Vonthein, Jane Tereszczuk, Arne Schreiber, Sebastian Liebau, Stefan Huttenlocher, Detlef Imhoff, Panagiotis Balermpas, Christian Keller, Kathrin Dellas, Rene Baumann, Claus Rödel, Guido Hildebrandt, Klaus-Peter Jünemann, Alex S. Merseburger, Alan Katz, Andreas Ziegler, Oliver Blanck, Jürgen Dunst

**Affiliations:** 1 Klinik für Strahlentherapie, Universitätsklinikum Schleswig-Holstein, Kiel, Germany; 2 Klinik für Strahlentherapie, Universitätsklinik für Medizinische Strahlenphysik, Pius Hospital, Oldenburg, Germany; 3 Universität zu Lübeck, ZKS, Lübeck, Germany; 4 Institut für Medizinische Biometrie und Statistik, Universitätsklinikum Schleswig-Holstein, Lübeck, Germany; 5 Saphir Radiochirurgie Zentrum Norddeutschland und Frankfurt am Main, Güstrow, Germany; 6 Klinik für Strahlentherapie, Universitätsklinikum Frankfurt, Frankfurt, Germany; 7 Klinik für Strahlentherapie, Universitätsmedizin Rostock, Rostock, Germany; 8 Klinik für Urologie, Universitätsklinikum Schleswig-Holstein, Kiel, Germany; 9 Klinik für Urologie, Universitätsklinikum Schleswig-Holstein, Lübeck, Germany; 10 Flushing Radiation Oncology Services, New York, USA; 11 Long Island Radiation Therapy, New York, USA; 12 School of Mathematics, Statistics and Computer Science, University of KwaZulu-Natal, Pietermaritzburg, South Africa; 13 Department for Radiation Oncology, University Clinic Copenhagen, Copenhagen, Denmark; 14 Department of Radiation Oncology, Karl Lennert Cancer Center, University Medical Center Schleswig-Holstein, Campus Kiel, Arnold-Heller-Straße 3, Haus 50, D-24105 Kiel, Germany

**Keywords:** Clinical trial, Localized prostate cancer, Extreme hypo-fractionation, Robotic Radiosurgery, Stereotactic body radiation therapy, CyberKnife

## Abstract

**Background:**

For prostate cancer treatment, treatment options with minimal side effects are desired. External beam radiation therapy (EBRT) is non-invasive, standard of care and delivered in either conventional fractionation over 8 weeks or with moderate hypo-fractionation over about 5 weeks. Recent advances in radiotherapy technology have made extreme hypo-fractionated stereotactic body radiation therapy (SBRT) of prostate cancer feasible, which has not yet been introduced as a standard treatment method in Germany. Initial results from other countries are promising, but long-term results are not yet available. The aim of this study is to investigate feasibility and effectiveness of SBRT for prostate cancer in Germany.

**Methods/design:**

This German bi-center single group trial (HYPOSTAT) is designed to evaluate feasibility and effectiveness, as measured by toxicity and PSA-response, respectively, of an extreme hypo-fractionated SBRT regimen with five fractions of 7 Gy in treatment of localized low and intermediate risk prostate cancer. The target volume includes the prostate with or without the base of seminal vesicles depending on risk stratification and uncertainty margins that are kept at 3–5 mm. SBRT treatment is delivered with the robotic CyberKnife system, which was recently introduced in Germany. Acute and late toxicity after one year will be evaluated according to Common Terminology Criteria for Adverse Events (CTCAE v. 4.0), Radiation Therapy Oncology Group (RTOG) and International Prostate Symptom Score (IPSS) Scores. The quality of life will be assessed before and after treatment with the EORTC QLQ C30 questionnaire. Hypothesizing that the proportion of patients with grade 2 side effects or higher is less or equal than 2.8%, thus markedly lower than the standard EBRT percentage (17.5%), the recruitment target is 85 patients.

**Discussion:**

The HYPOSTAT trial aims at demonstrating short term feasibility of extreme hypo-fractioned SBRT for the treatment of prostate cancer and might be used as the pilot study for a multi-center multi-platform or for randomized-controlled trials comparing conventional radiotherapy with SBRT for localized prostate cancer in the future. The study concept of patient enrollment, follow up and evaluation by multiple public university clinics and actual patient treatment in dedicated private radiosurgery practices with high-tech radiation equipment is unique for clinical trials.

**Study status:**

The study is ongoing and currently recruiting patients.

**Trial registration:**

Registration number: NCT02635256 (clinicaltrials.gov). Registered 8 December 2015.

## Background

### General treatment strategy for localized prostate cancer

Prostate cancer is the most frequently diagnosed malignant tumor and the third most frequent reason for men’s death in Europe [1]. Current therapy procedures for localized prostate cancer include active surveillance, prostatectomy and radiation therapy, but the optimal therapy regime remains unclear.

Compared to other cancers, prostate cancer is characterized by three specific features: the progression rate is slow, the patient population is old with a median age at diagnosis of approximately 75 years and a specific tumor marker — the prostate specific antigen (PSA) is available to monitor progression even at an early stage [2–5]. Owing to these facts, ‘active surveillance’ is more and more considered as an alternative treatment option for localized low- to intermediate risk prostate cancer [6–8]. During active surveillance, digital rectal exams and annual biopsies are performed and PSA levels are checked regularly and therapeutic intervention is only recommended in case of disease progression (e.g., as measured by increased PSA levels). Some studies have confirmed that early intervention with radical prostatectomy may have no advantage for most patients when compared to active surveillance [9–11].

Local therapies, such as radical prostatectomy or radiation therapy, either in form of external beam radiotherapy (EBRT) or brachytherapy or a combination of both, are indicated if active surveillance is impossible or unwanted or in case of documented disease progression. Radiation therapy is, with respect to tumor control, at least equivalent to radical surgery for all patients with localized disease. The range of side effects is different with the major problems being incontinence after prostatectomy and rectal complications after radiotherapy. However, the overall frequency of side effects seems to be similar [12–18]. The choice of local therapy method, therefore, mainly depends on the patient’s preference and possible contraindications.

Overall, the above mentioned treatment options (active surveillance and local therapy) are compared in several clinical trials: the most recent publication coming from the ProtecT trial [19]. The study group published its 10 year results in 2016 and found that overall survival was not different in the active surveillance and local therapy group; however there seems to be a benefit for local therapy in terms of disease progression and development of metastases [19]. In Germany, a large randomized study (PREFERE) was initiated in 2013 with the objective to compare the different treatment procedures including active surveillance, prostatectomy, brachy-therapy and EBRT for low risk prostate cancer. PREFERE was the largest oncological clinical trial in Germany and financially supported by the German Cancer Aid and includes patients younger than 75 years at diagnosis with a PSA value ≤ 10 ng/ml and a Gleason score ≤ 7a (3 + 4). Approximately 7300 patients were planned to be treated, but the study was recently closed.

### External beam radiotherapy

EBRT for low/intermediate risk prostate cancer is generally carried out with conformal- (3D-CRT) or intensity- and volumetric-modulated radiotherapy (IMRT and VMAT) techniques delivered in conventional fractionation [20–23]. The target organ (prostate, possibly also the seminal vesicles or a part of them) is irradiated with single doses of 1.8–2.0 Gy daily for 5 days a week up to a total dose of 72–78 Gy [20–23]. There is a clear dose-response relationship between tumor control rates and total irradiation doses [24, 25]. However, better local control has not resulted in increased survival. Moreover, almost all studies have also shown an increase of late toxicity at higher doses [24, 25]. For example, a large randomized-controlled trial found that 39% of all patients developed genitourinary (GU) toxicity, and 32% had grade 2 gastrointestinal (GI) toxicities after 4.2 years of follow up [24]. After 1 year, approximately 17.5% of patients had grade 2 late complications in the GU system and 10% developed toxicity in the GI system.

### Rationale for treating localized prostate cancer using hypo-fractionation

In general, conventional fractionation for EBRT with daily doses of 1.8–2.0 Gy is based on the assumption that the cells of the surrounding healthy organs have a lower proliferation rate than the tumor cells and are therefore less reactive to small radiation doses. The cell survival of an organ or tumor after irradiation is generally described by the α/β-value [26] — high proliferating organs or tumors have α/β-values in the order of 10 Gy and very slow proliferating organs or tumors have α/β-values in the order of 2 Gy.

Recent data from various prospective studies suggest a low fractionation sensitivity of prostate cancer [26, 27]. Currently, the α/β-value of prostate cancer is estimated to be approximately 1.5 Gy; thus, lower than the currently estimated α/β-value for late rectal complications, which is approximately 3.0 Gy [28, 29]. This difference in fractionation sensitivity suggests that an advantage might be expected from hypo-fractionated treatment regimens, either with regard to increased local control, reduced late side effects or both. Interestingly, published randomized trials that used moderately hypo-fractionated regimens (e.g., 22 vs. 40 fractions) showed only similar results with regard to biochemical control and even slightly worse side effects compared to standard fractionation [30, 31]. A comprehensive review on the topic of moderate hypofraction for prostate radiotherapy was recently published by a German group [32].

### Treatment results with extreme hypo-fractionation

Following the concept of the assumed radiobiology of prostate cancer to the end, several studies in the USA investigated extreme hypo-fractionated regimens, called stereotactic body radiation therapy (SBRT) or sometimes also called radiosurgery. These studies used 5 fractions, and total doses of 35–37 Gy have been reported recently [33–45]. Up to now, more than 1500 patients with 5 years of follow up were treated in 18 different feasibility studies. Most of the studies used robotic SBRT with the CyberKnife system (Accuray Inc., USA) and active fiducial-based tracking because of well-known prostate motion during treatment [46, 47]. Due to relevant prostate movements during treatment, techniques with image-guidance and motion compensation seem mandatory and a widespread use of extreme hypo-fractionation is currently not recommended [48–51].

In brief, these studies showed excellent biochemical control rates after 1–5 years in the range of 93–100% for low and intermediate risk prostate cancers (PSA value < 20 ng/ml and Gleason score ≤ 7). These studies also demonstrated a low frequency of late side effects with a trend towards more severe side effects if a total dose of 35 Gy in 5 fractions were exceeded. Mild side effects (grade 2) with 35 Gy and 36.25 Gy in 5 fractions were 4% and 10% in the GU system, respectively, and 2% and 4% in the GI system, respectively. Serious GU side effects (grade 3) occurred in only 2% of patients treated with 36.25 Gy. These data are in favor of SBRT when compared to the results of standard fractionated EBRT [24, 25, 35, 37]. Recently, the first results of the Scandinavian HYPO-RT-PC trial comparing 42.7 Gy in 7 fractions versus 78 Gy in 39 fractions confirmed this hypothesis [52] and the randomized controlled HEAT (USA) and PACE (UK) trials are actively recruiting patients.

As the treatment of prostate cancer with extreme hypo-fractionated SBRT has not yet been introduced as common practice in Germany and long-term follow-up data are not yet available internationally, the German Society for Radiation Oncology (DEGRO) and the National Radiation Protection Authority (BfS, Bundesamt fuer Strahlenschutz) have issued a strict recommendation to only treat prostate cancer with extreme hypo-fractionation in prospective clinical trials. This body of work represents the first and currently only clinical trial for hypo-fractionated SBRT for localized prostate cancer (HYPOSTAT) in Germany.

## Methods/design

### General study setting

At the time of study protocol initiation of HYPOSTAT in 2012, two main circumstances were found for Germany with respect to prostate cancer treatment: 1) the PREFERE trial was widely supported and conducted by many institutions, and 2) the CyberKnife, from which most prostate treatment data for hypo-fractionated SBRT originated, was only available in a limited number of centers, most being privately owned. It was therefore intended to initiate a clinical trial that included the private CyberKnife centers as well as excluded patients eligible for the PREFERE trial. The current setting in amendment IV from May 2016 of the HYPOSTAT study (protocol number ZKS-121-003) stipulates that patients are enrolled and followed after treatment by public university medical centers and treated by the CyberKnife system in two private practices. At the time being, this is a unique setting in clinical trial design.

### Study design

The HYPOSTAT study is designed as a prospective observational trial. The hypothesis is that image-guided SBRT with 35 Gy in 5 fractions (7 Gy per fraction) is feasible in the treatment of prostate cancer. The primary endpoint is toxicity to the GI and GU system at 1 year after treatment assessed according to the Common Terminology Criteria for Adverse Events (CTCAE v. 4.0) and Radiation Therapy Oncology Group (RTOG) Scores. Secondary endpoints are PSA-failure-free survival, relapse-free survival, urological function assessed by the International Prostate Symptom Score (IPSS), and Quality of life (QoL) assessed before and after treatment with the European Organization for Research and Treatment of Cancer (EORTC) Quality of Life Questionnaire (QLQ) C30 and the Patient Oriented Prostate Cancer Utility Scale (PORPUS) questionnaire.

### Patient inclusion criteria

- Histologically confirmed, locally confined prostate cancer
- Completed staging according to the National Guidelines
- Gleason score ≤ 7
- PSA < 15 ng/ml
- Prostate volume < 80 cm^3^
- IPSS-Score ≤ 12
- Age > 75 years or Age 70–75 years and either PSA > 10 ng/ml and/or Gleason score = 7b and/or Gleason score = 7a with > 33% positive biopsy cores and/or cT > 2a and/or prostate volume > 60 cm^3^
- Informed consent

### Patient exclusion criteria

- Treatment in the PREFERE trial possible
- Previous pelvic radiotherapy
- Contraindication against the implantation of fiducial gold markers
- Immunosuppressive therapy
- Treatment relevant co-morbidities
- Patient’s inability to understand or comply with the procedures

### Treatment planning and delivery

Treatment will be carried out with a robotic CyberKnife system (Accuracy Inc., USA) using real-time tracking. Three to four fiducial markers will be necessary to identify the location of the prostate during treatment. The distance between two fiducial markers should be ≥ 2 cm. Implantation of fiducial markers should be completed at least 5 days before treatment-planning computed tomography (CT) to reduce the post-implantation migration of the fiducial marker. During planning CT, and on any treatment day, the use of an enema is advised to reduce the maximum rectal diameter. A bladder catheter is not necessarily needed, but a consistent and constant bladder filling of 20–30 ml during planning CT and treatment is recommended.

Gross tumor volume (GTV) is defined as the prostate on the planning magnetic resonance imaging (MRI) for patients with a low-risk profile and as the prostate with the bases of seminal vesicles (1 cm proximal) for all other patients. The clinical tumor volume (CTV) is defined as GTV plus 1–2 mm for patients with low-risk prostate cancer and plus 2 mm for all other patients. The planning target volume is defined as CTV plus an anisotropic margin of 1 mm posterior into the direction of the rectum and 3 mm in all other directions. Organs at risk (OAR) are rectum, bladder, urethra (if identifiable), neurovascular bundles (if identifiable), penile bulb, small bowel, femoral heads and the skin (5 mm thick). The rectum is defined from the inferior level of the anus to the recto-sigmoid junction to give a length of approximately 12 cm.

All patients will be treated with 35 Gy in 5 fractions (7 Gy per fraction) prescribed to the 80–85% isodose (maximum dose = 100%) covering the PTV to 95% (PTV_35Gy_ ≥ 95%). OAR dose limitations (Table 1) are specified according to published guidelines [33–45, 53–56] and the urethral dose reduction technique will be used to minimize risks of side effects [57]. Patients will be treated every second day with a minimum of 36 h between two fractions. The treatment time shall be 1.5 to 2 weeks.

**Table 1:** Dose limits for organs at risk relevant for prostate radiosurgery

Organ at risk	Dose limit
Rectum	D_max_ < 38 Gy V_36Gy_ < 1 cm^3^ & < 5% V_29Gy_ < 15 cm^3^ & < 20% V_18Gy_ < 25 cm^3^ & < 50%
Bladder	D_max_ < 38 Gy V_36Gy_ < 10 cm^3^ & < 10% V_18Gy_ < 40%
Urethra	D_max_ < 44 Gy
Penile Bulb	V_30Gy_ < 3 cm^3^ & < 50%
Femoral Heads	V_30Gy_ < 10 cm^3^ V_14,5Gy_ < 5%
Testicle	To be blocked for passing beams
Small Bowel	V_30Gy_ < 1 cm^3^ V_18Gy_ < 5 cm^3^
Neurovascular Bundle	V_38Gy_ < 50%
Skin (5 mm)	D_max_ < 30 Gy

*D*_*max*_ *=* Maximum Dose, *V*_*xGy*_ *=* Volume receiving X Gy or more

### Follow-up assessments

The primary endpoint will be the feasibility of extreme hypo-fractionated SBRT in the treatment of prostate cancer assessed 1 year after treatment, as all of the acute and certain intermediate toxicity will be observed in this period. Follow-up visits will occur at 4–6 weeks, 3 months (± 1 week), 6–9 months and 12–15 months after the last day of irradiation. During follow up, side effects will be documented (if any) and the PSA value (at least twice), IPSS and QoL using the PORPUS questionnaire will be documented. The course of the PSA value will be used to declare biochemical failure according the Phoenix definitions. In case of failure to identify disease progression by assessing the PSA value, progression events are defined as radiologically documented disease progression using Response Evaluation Criteria In Solid Tumors (RECIST) Criteria (Revised Guidelines, Version 1.1, 2009). QoL is assessed using the EORTC QLQ C30 questionnaire at the last follow-up visit.

The reasoning for this short follow-up time was due to the nature of the study design being a feasibility trial with a single technique which would serve as the basis for a subsequent multi-institutional multi-platform trial with long term follow-up. I.e., we did not want to overly delay such long term multi-institutional multi-platform study and decided to finish the initial feasibility trial as soon as possible. For long-term evaluation, patients will of course be followed for at least 5 years according to the standard practice for radiotherapy in Germany which additionally will include treatment relevant efficacy, side effects, IPSS and QoL assessments; however, this will not be part of the study protocol.

### Sample size calculation

Based on available data and radiobiological calculations, the frequency of severe toxicity (grade 2 or higher) is expected to be approximately 2.8% for GU and 1.1% for GI toxicity. Eighty-five and fifty-four patients are required to reject proportions of 17.5% and 10% with 78% and 88% power at two-sided significance levels < 5% with the exact binomial test using a Bonferroni-Holm procedure. The aim is therefore to recruit 85 patients in this trail.

### Trial duration and criteria for early study termination

Patient accrual is estimated to be finished within 2 years, with a follow-up period of 1 year after last patient in. The clinical trial should be concluded 3 years after first inclusion. Early termination of the trial will occur if the toxicity of this extreme hypo-fractionated SBRT is determined to be unacceptable (≥ grade 3 in more than four of the first ten patients for acute or more than 20% of all patients for chronic side effects). The study can be terminated early, if a) the hypothesis of the study can be answered by new data, b) the understanding of the radiobiology for prostate cancer is markedly altered by new data, or c) the recruiting target is not met during the recruitment phase.

### Ethical and legal considerations

The HYPOSTAT study protocol was reviewed by the ‘Independent Expert Committee of the DEGRO’ and an approval from the National Radiation Protection Authority (BfS) for conducting the study was deemed necessary. The study was approved by the BfS (reference number Z5–22463/2–2013-031) and by the independent ethics committee of the University of Luebeck (reference number 13–052) and, subsequently, by the ethics committees responsible for the participating clinics and institutions (University of Kiel, University of Frankfurt and University of Rostock). The HYPOSTAT study is accredited by the Radiation Oncology Working Group (ARO) of the German Cancer Society. The registration number at clinicaltrials.gov is NCT02635256. The study is monitored and audited by the center for clinical trials (ZKS Luebeck, Germany, protocol number ZKS-121-003). Sponsor of the study is the University Medical Center Schleswig Holstein, and the study is partly financed by the Dr.-Rurainski-Stiftung (Ettlingen, Germany) and the Saphir Medical Engineering Group GmbH (Guestrow, Germany).

## Discussion

The HYPOSTAT trial aims at demonstrating short term feasibility of extreme hypo-fractioned SBRT for the treatment of prostate cancer and might be used as the pilot study for a multi-center multi-platform or for randomized-controlled trials in Germany comparing conventional radiotherapy with SBRT for localized prostate cancer in the future. The study concept of patient enrollment, follow-up and evaluation by multiple public university medical centers and actual patient treatment in dedicated private radiosurgery practices with high-tech radiation equipment is unique for clinical trials.

## Abbreviations

BfS: Bundesamt fuer Strahlenschutz (National Radiation Protection Authority)
CT: Computer Tomography
CTCAE: Common Terminology Criteria for Adverse Events
CTV: Clinical Target Volume
DEGRO: Deutsche Gesellschaft fuer Radioonkologie (German Society for Radiation Oncology)
EBRT: External Beam Radiation Therapy
EORTC: European Organization for Research and Treatment of Cancer
GI: Gastrointestinal
GTV: Gross Target Volume
GU: Genitourinary
HYPOSTAT: Hypofraktionierte Strahlenchirurgie bei lokal begrenztem Prostatakarzinom (Hypo-fractioned radiosurgery for localized prostate cancer)
IMRT: Intensity Modulated Radiation Therapy
IPSS: International Prostate Symptom Score
MRI: Magnetic Resonance Imaging
NCI: National Cancer Institute
OAR: Organ at Risk
PORPUS: Patient Oriented Prostate Cancer Utility Scale
PSA: Prostate Specific Antigen
PTV: Planning Target Volume
QLQ: Quality of Life Questionnaire
QoL: Quality of Life
RECIST: Response Evaluation Criteria In Solid Tumors
RTOG: Radiation Therapy Oncology Group
SBRT: Stereotactic Body Radiation Therapy
VMAT: Volumetric Modulated Radiation Therapy
